# Current therapeutic strategies and available research models for investigating hypertrophic scars

**DOI:** 10.3389/fphar.2026.1887685

**Published:** 2026-09-09

**Authors:** Alexa Chu, Suneel Kumar, Francois Berthiaume

**Affiliations:** Department of Biomedical Engineering, Rutgers, The State University of New Jersey, Piscataway, NJ, United States

**Keywords:** collagen, fibrosis, hypertrophic scar, remodeling, scar, spinal cord injury

## Abstract

Abnormal scarring, such as hypertrophic and keloid scars, are a result of pathological skin wound healing, and can reduce quality of life through psychological factors, persistent pain, and reduced mobility. Under physiological conditions, wounds proceed sequentially through the hemostatic, inflammatory, proliferative, and remodeling phases of healing. However, dysregulated wound healing can overstimulate fibrotic activity, resulting in the development of hypertrophic or keloid scars. These scars present a significant clinical challenge, as hypertrophic scars develop in more than 70% of burn survivors with a 40%–94% incidence rate following surgery. While hypertrophic and keloid scars are a common health concern, treatment options are limited due to the complex nature of pathological scar development. Furthermore, the development of new therapeutics is complicated by limitations in research models mimicking scars *in vitro* and *in vivo*. We present a review of the available therapeutic strategies and clinical data addressing fibrotic scars, *in vitro* and *in vivo* models, and methods used to study scars. We then summarize recent clinical trial data addressing hypertrophic scars. Lastly, we discuss the translatability of hypertrophic scar research to other pathological scars, such as mechanically weak scars and scars from spinal cord injury-induced pressure wounds that increase the risk of wound recurrence after healing in certain types of wounds. Findings identify several early inflammatory signaling targets to investigate for the improvement of hypertrophic scarring.

## 1 Introduction

Hypertrophic and keloid scar formation are caused by dysregulated wound healing and excessive fibrotic activity (Lin and Lai, 2024). The resultant scar is often pruritic, painful, and in severe cases causes significant movement restriction and disfigurement to the patient (Mony et al., 2023; Carswell and Borger, 2025). The occurrence rate of hypertrophic and keloid scars is high, as 30%–90% of individuals will develop such scars, particularly in deep surgical wounds and burns (Barone et al., 2021). While fibrotic scarring is a complex physiological issue, risk factors for hypertrophic scars include young age, dark skin pigmentation (Type IV-VI Fitzpatrick skin type), high BMI and prior pro-inflammatory conditions, and deep wounds and burns in high-movement areas (Thompson et al., 2013; Carswell and Borger, 2025). Given the various factors contributing towards the formation of hypertrophic scars, treatment often involves multiple preventative and post-scarring measures. However, despite several treatment options (excision, intralesional injections, lasers, etc.), recurrence of hypertrophic and keloid scars remains a concern (Anderson et al., 2017; Rimmer et al., 2023). Furthermore, research models for hypertrophic scars are limited due to challenges in accurately simulating the scar *in vitro* and *in vivo*.

This review covers the development of pathological scars, the molecular and biological drivers of hypertrophic scarring, clinical data and available treatments, and the current methods and models used to study hypertrophic scars *in vitro* and *in vivo*. Lastly, we discuss the translatability of hypertrophic scar research for use in cases of other pathological scars such as mechanically weak scars with lower fibrotic activity that increase susceptibility to wound recurrence, such as in the case of pressure wounds after traumatic central nervous system (CNS) injury (Paker et al., 2018; Kumar et al., 2021).

### 1.1 Normal and pathological scar physiology

#### 1.1.1 Normal scar formation

**FIGURE 1:**
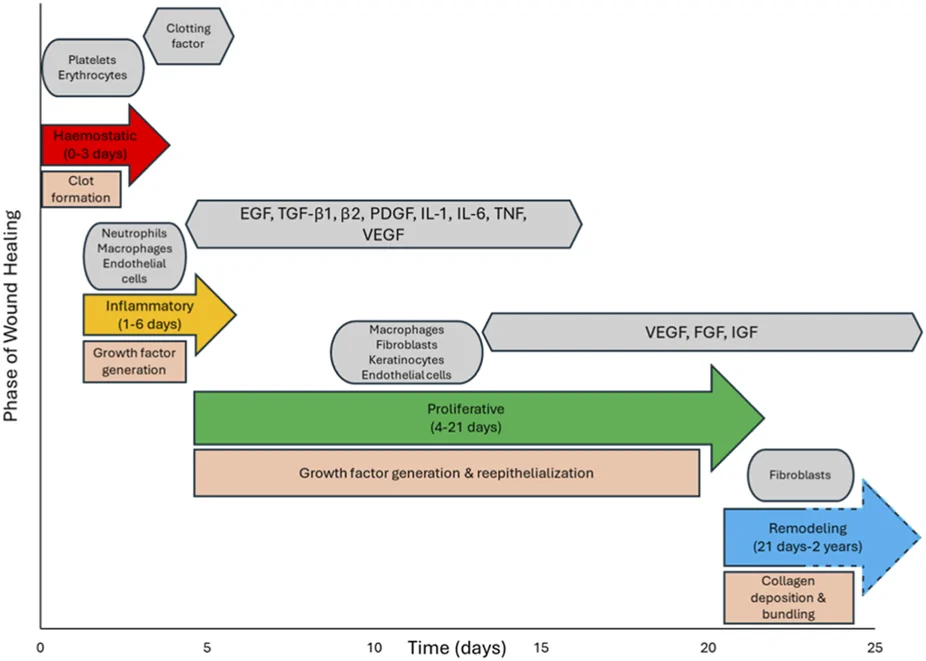
Phases of normal physiological wound healing with respect to active cell types and cytokines released. Under normal wound healing conditions, the completion of the remodeling phase results in the formation of a healthy scar with physiological collagen bundling and alignment. Pathological wound healing can result in overexpression of inflammatory and pro-fibrotic cytokines during the inflammatory and proliferative phases of healing, resulting in hypertrophic scarring (Chu et al., 2025). All abbreviated cytokines are mentioned in main text.

The normal physiological phases (hemostatic, inflammatory, proliferative, and remodeling) of wound healing initiate immediately post-injury, during which hemostasis and clot formation occur (Figure 1). Following the hemostatic phase is the inflammatory phase, where the primary cells acting are neutrophils and macrophages, releasing cytokines and growth factors into the wound space, thereby recruiting keratinocytes, fibroblasts, and endothelial cells in the proliferative phase. Subsequently, endothelial cells initiate angiogenic activity and revascularize the space, and keratinocytes and fibroblasts re-epithelialize the wound boundary. The final phase of wound healing is the remodeling phase, which includes collagen fiber bundle formation and organization, typically resulting in a normal scar (Almadani et al., 2021; Wallace et al., 2025).

### 1.2 Pathological scar formation

A significant percentage of remodeled scars develop into hypertrophic or keloid scars due to excessive fibroproliferation and aberrations in the wound healing process. For example, during the hemostatic phase, platelets release pro-fibrotic growth factors such as platelet-derived growth factor (PDGF), vascular endothelial growth factor (VEGF), transforming growth factor (TGF-β), and tumor necrosis factor-alpha (TNF-α) which contribute towards a high-density blood clot (Werner and Grose, 2003; Nam and Kim, 2018; Mony et al., 2023). In cases of wounds with persistent or chronic inflammation, key inflammatory cells such as macrophages and neutrophils secrete pro-fibrotic growth factors which are compounded during the subsequent proliferative phase (Figure 1) (Chu et al., 2025). M2 macrophages specifically have been identified for their role in stimulating differentiation of fibroblasts into myofibroblasts through the release of TGF-β, and PDGF-CC (Glim et al., 2013; Hesketh et al., 2017; Wang L. et al., 2020). Myofibroblasts are directly responsible for remodeling and contracting the collagen and extracellular matrix (ECM), and are the primary driver of Type I and Type III collagen release and Type III to Type I strengthening (Darby et al., 2016; Schuster et al., 2023). As such, irregular or excessive myofibroblast activity can lead to fibrosis and scarring (Peña and Martin, 2024). Following the inflammatory phase, the release of pro-fibrotic growth factors (PDGF, VEGF, FGF) is increased during the proliferative phase, thereby stimulating angiogenesis and re-epithelialization (Sinno and Prakash, 2013). Pathological wounds have been characterized to have excessive vascular leakage and chronically increased vascular permeability during the inflammatory and proliferative phases, resulting in the uptake of inflammatory cytokines and clotting factors (Wichaiyo, 2025). It was found that pathological stroma formations, particularly in cases of wounds with delayed hypersensitivity reactions, often have hyperpermeability of dermal vasculature to plasma proteins and clotting factors as well as abundant deposits of dermal cross-linked fibrin (Dvorak, 2021). More specifically, the hyperpermeability in pathological wounds leads to the uptake of plasma proteins such as fibrinogen and other clotting factors, resulting in fibrinogen-to-fibrin crosslinking (Dvorak, 2021).

There is significant clinical evidence tying over-vascularization to fibrotic scarring. Stereological analysis of normal and fibrotic tissue explants showed significantly higher vascular density and dilation in hypertrophic, and keloid scars relative to normal tissue (Amadeu et al., 2003). Hypertrophic vasculature has been clinically characterized to have a dendritic pattern of growth with large bore vessels, often growing outside the boundaries of the original wound (Shakespeare and Jones, 1992). As such, it has been shown that fibrotic scarring is directly related to hyperactive vascular response during abnormal wound healing, and limiting the angiogenic activity during the proliferative phase is a potential therapeutic target to induce scar regression (Yuan et al., 2023).

The overabundance of myofibroblasts alters the synthesis of ECM and collagen bundle formation by over-depositing Type III and Type I collagen during the remodeling phase of wound healing (Mony et al., 2023). This is mediated through macrophage-mediated TGF-β signaling, exacerbated by a decreased level of metalloproteinases (MMPs) which degrade the ECM and excess collagen (Hinz et al., 2019; Younesi et al., 2024; Jia et al., 2025).

It has also been shown that mechanotransduction signaling contributes to hypertrophic scarring. Keloid and hypertrophic scars are characterized as having a propensity to form in anatomical areas that experience increased stiffness and mechanical forces (Berry et al., 2023). The pathogenesis of keloid scars, including their shape, tends to follow areas of highest tensile force (Ogawa et al., 2012; Berry et al., 2023). External mechanical forces on the wound translate to increased cellular inflammatory signaling and TGF-β expression, known as mechanotransduction. The increased release of TGF-β upregulates myofibroblast contractility and fibroblast contractile markers such as alpha-smooth muscle actin (α-SMA) (Arora et al., 1999; Vaughan et al., 2000; Thannickal et al., 2003; Dabiri et al., 2006; Berry et al., 2023).

In summary, the formation of hypertrophic and keloid scars is driven by a compounding of pro-fibrotic factors that remain unchecked in pathological wound healing conditions in addition to mechanotransduction from external forces on the wound.

### 1.3 Differentiating hypertrophic and keloid scars

Hypertrophic and keloid scars are both forms of fibrotic scarring, with similar pathogenesis and pathophysiology. Keloid scars definitionally grow beyond the initial scar boundary, unlike hypertrophic scars (Limandjaja et al., 2021). Hypertrophic and keloid scars are thought to have shared etiology, generally stemming from a strong inflammatory response and exacerbated by stretch-mediated signaling in the wound (Carswell and Borger, 2025). Keloid scars are suggested to form partly from genetic predisposition, as the epidemiologic prevalence of keloids are much higher in people of color (Glass, 2017; Tchero, 2020). Hypertrophic scars are not as strongly linked to genetic predisposition, but mechanical tension and stretching in the affected area (Butzelaar et al., 2016; Carswell and Borger, 2025). The formation of both hypertrophic and keloid scars is caused by excessive inflammatory signaling, namely, dysregulated TGF-β expression which alters collagen production in the scar (Tchero, 2020; Mony et al., 2023; Carswell and Borger, 2025). A differentiating factor between the two scar types is that while both scars have increased collagen fibers, the fibers in hypertrophic scars are organized parallel to the dermis while keloid scars are less organized and have no clear orientation (Carswell and Borger, 2025). Clinically, the management and treatment strategies for hypertrophic and keloid scars are the same and are discussed in the following section (Limandjaja et al., 2021).

## 2 Clinically available therapies for hypertrophic scars

Conventional treatments for hypertrophic scarring are primarily early intervention strategies to prevent the formation of fibrotic scarring and post-scarring treatments intended to disrupt or remove the scar tissue entirely (Mony et al., 2023).

### 2.1 Early intervention and preventative treatment

Early preventative therapies involve regulating normal wound healing and stabilizing the wound to limit mechanical stretch-stimulation of hypertrophic scarring. In the case of surgical wounds, incisions should be made following the natural orientation of the patient collagen fibers to minimize fibrosis and abnormal scarring (Carmichael, 2014; Mokos et al., 2017). Furthermore, suture closure techniques can be utilized to reduce the amount of tension on the wound closure (Ogawa et al., 2011). Non-surgical wounds should be managed to promote normal wound healing through proper cleaning and local anti-bacterial treatment to reduce excessive inflammatory response and pro-fibrotic activity (Nischwitz et al., 2020). Upon wound closure and re-epithelialization, silicone sheets and gels are firstly recommended to provide a hydrated barrier and prevent inflammation and scarring (Bleasdale et al., 2015; Moortgat et al., 2019). For severe wounding, compression garments may also be recommended to stabilize the tissue and reduce capillary perfusion (Macintyre and Baird, 2006; Mokos et al., 2017; Mony et al., 2023). Vascular, ablative, and non-ablative laser treatments are also considered a first-line therapy for treating traumatic scars as they have the ability to eliminate over-vascularization in the wound and reduce fibrosis. However, laser treatment can result in hyperpigmentation particularly in darker skin phototypes (Kuehlmann et al., 2019; Seago et al., 2020; Won et al., 2022). As such, these therapeutic strategies generally involve wound healing management and stabilizing the wound closure to minimize stretch-mediated hypertrophic scarring.

### 2.2 Post-scarring management

Second-line management of hypertrophic scarring can include surgical excision of the fibrotic tissue, though resection of hypertrophic and keloid scars results in high recurrence rates and should be combined with additional therapies (Ogawa, 2022). Due to high recurrence rates and surgical risk, excision alone is not often recommended and other secondary treatment options are preferred such as laser or microneedling treatments (Ogawa, 2022; Mony et al., 2023). Microneedling is a minimally invasive technique which has been shown to disrupt the local scar tissue and collagen fibers, stimulating the release of MMP-9 which can degrade excessive collagen, though there is limited supporting clinical trial data available (El Mofty et al., 2017; Juhasz and Cohen, 2020; Vijaya Lakshmi et al., 2020). Corticosteroids administered through intralesional injection have a strong anti-inflammatory effect on hypertrophic scarring, and restrict local vasculature (Abedini et al., 2018; Ogawa, 2022). While corticosteroid injection is an effective treatment for reducing hypertrophic scarring, it can atrophy the skin or result in hyperpigmentation of the affected area (Sheng et al., 2023).

### 2.3 Limitations of available treatments

The overall therapeutic landscape for hypertrophic scarring varies from topical agents (silicone gel, sheets, anti-inflammatory and anti-bacterial creams), scar compression and stabilization techniques (strategic suturing, compression garments), laser and/or excisional procedures (ablative and non-ablative), microneedling procedures, and intralesional corticosteroid injections. There is no singular gold-standard treatment for hypertrophic scarring, as recommended therapeutic strategies span the entirety of the wound healing process, and often multiple treatment techniques are clinically recommended. The limitations of the clinically available treatments are summarized (Table 1).

**TABLE 1:** Clinically available treatments for hypertrophic scarring, including primary mechanism of action, advantages, and disadvantages of therapeutic option.

Treatment	Mechanism of action	Advantages	Disadvantages	References
Silicone-based products	Occlusion and hydration barrier, reduces local inflammation	Non-invasive, generally well tolerated	Works best when worn long-term, should be in addition to another treatment	Bleasdale et al. (2015), Moortgat et al. (2019)
Suture techniques	Reduces tension on wound closure	Simple alternative method of wound closure, proactively reduces stretch-induced hypertrophic scarring	Surgical procedure, invasive, should be in addition to another treatment	Ogawa (2020), Shin et al. (2025)
Compression tapes and garments	Reduces tension and limits movement around wound closure, reduces capillary perfusion	Non-invasive, generally well tolerated	Works best when worn long-term, can be uncomfortable, should be in addition to another treatment	Harris et al. (2024)
Laser	Eliminates local microvasculature, ablative and non-ablative options can reduce perfusion to tissue or remove fibrosis	Minimally invasive, effective second-line treatment for closed wounds to reduce hypertrophic scarring	Expensive, can cause hyperpigmentation in darker skin types, erythema, can cause pain, should be in addition to another treatment	Kuehlmann et al. (2019), Seago et al. (2020), Won et al. (2022)
Microneedling	Disrupt collagen fibers in hypertrophic scar, induce release of MMP-9	Minimally invasive, effective second-line treatment for closed wounds to reduce hypertrophic scarring, less likely to cause hyperpigmentation in darker skin types	Expensive, can cause erythema and pain, should be in addition to another treatment	Juhasz and Cohen (2020), Vijaya Lakshmi et al. (2020)
Excision/resection	Remove hypertrophic scar tissue	Can remove larger scar tissues	Invasive, expensive, requires surgery and potential anesthesia. High recurrence rate without additional treatment	Ogawa (2022), Tomtschik et al. (2024)
Corticosteroid injection	Reduce inflammation in hypertrophic scar tissue, vasoconstriction and limit perfusion to scar	Effective in reducing height of hypertrophic scarring	Invasive, can be expensive, can cause pain or discomfort. Risk of skin atrophy and hyper/hypopigmentation	Abedini et al. (2018), Ogawa (2022)

Current clinical guidelines include early interventions such as lower tension suture techniques, compression garments, and silicone-based occlusives to prevent or limit the formation of hypertrophic scars (Monstrey et al., 2014; Shirakami et al., 2020). Second-line intervention therapies also include intralesional corticosteroid injections, which are supported by multiple controlled clinical trials and have been clinically in use for many years (Shin et al., 2025). Additional clinically supported and recommended interventions are laser treatments, and combinations of the aforementioned treatments are often performed as they have been thoroughly investigated and found to improve hypertrophic scar outcomes (Monstrey et al., 2014; Shin et al., 2025). Thus, suture techniques, silicone-based occlusives, laser therapies, compression, and intralesional corticosteroid injections are well-supported by clinical data and are regularly employed in clinic as first-line interventions (Table 1). There is limited clinical evidence on emerging and investigational therapies such as cell penetrating asymmetric siRNA (cp asiRNA) treatments which interrupt connective tissue growth factor (CTGF) production, or interleukin-4 receptor subunit α (IL-4R α) and TGF-β inhibitors discussed in Section 3.1. While these emerging therapies are designed to address molecular targets and mechanisms of hypertrophic scarring (discussed in Section 4.1), more clinical evidence is needed to support the use of these emerging therapies.

It is noteworthy that none of the currently available therapies directly address the molecular targets responsible for the pathogenesis of hypertrophic scars. Pro-fibrotic and inflammatory markers such as IL-6, IL-8, TGF-β and signal transducer and activator of transcription 3 (STAT3) are known to regulate myofibroblast differentiation and the release of contractile markers, which impacts collagen remodeling behavior (Mony et al., 2023). Despite several inflammatory and fibrotic signaling pathways being identified in the development of hypertrophic scars, current therapeutic strategies seemingly center around mechanical disruption or removal of scar tissue (e.g., suture techniques and compression garments to reduce scar tension, or laser, microneedling, and excision which disrupt the collagen fibers or remove the tissue altogether). For further discussion of the relevant molecular targets, see Section 4.

Given the multi-mode nature of hypertrophic scarring, and their formation through a cascade of factors throughout the entirety of the wound healing process, multiple treatment options are generally recommended. It is apparent that there is no single therapeutic answer to the complex problem of hypertrophic scarring. Treatment of hypertrophic scars should take a multi-treatment approach to address contributing pro-fibrotic factors from the early wounding phase through closure and reepithelialization. Then, it is recommended to investigate other therapeutic targets to prevent and treat hypertrophic scarring.

## 3 Recent clinical trials on hypertrophic scars

### 3.1 Current clinical trial data

A review of clinical trials completed in the past 5 years was performed using ClinicalTrials.gov.

Search strings used were “hypertrophic scar”, “keloid scar” conditions. The search was conducted between April-May 2026, selecting any completed studies started by January 1, 2021. There were no search restrictions or exclusions on sex, age, or country. The search produced 27 studies, of which 8 studies pertaining to atrophic scars and acne scars were excluded as they were not relevant to fibrotic scarring.

The review of the recently completed clinical trial studies including sample size, trial phase, and outcomes is tabulated (Supplementary Table S1). To summarize, the clinical trial studies focus largely on the assessment of laser techniques, compression garments/tapes, intralesional corticosteroid injections, topical gels, and dressings in the treatment of hypertrophic and keloid scars.

Many of the intervention techniques studied (lasers, compression, negative pressure) involved preventing scarring through physical stabilization and compression or treating the scar by mechanically disrupting the collagen fibers and tissue structure. These interventions are consistent with the clinically available treatments discussed in Section 2. Of the 19 studies tabulated, 9 studies involved a drug-based intervention generally administered as an intralesional injection. Some drug interventions included general corticosteroids (triamcinolone acetonide), cholecalciferol (vitamin D), 5-fluorousil, and protescal gel barrier. However, three studies investigated drug mechanisms that interrupted specific molecular targets responsible for hypertrophic scarring. NCT04877756 tested OLX10010, which is a cp asiRNA treatment which interferes with the expression of CTGF (Lee, 2025). Furthermore, NCT05128383 tested Dupilumab, a human monoclonal antibody which inhibits the IL-4R α (Gade et al., 2026). Lastly, NCT04951869 used topical Pirfenidone to modulate the TGF-β pathway and therefore reduce the expression of pro-inflammatory and pro-fibrotic markers (Knighton et al., 2025). These particular drug interventions target specific markers and signaling activity which have been identified to cause the formation of hypertrophic scars. While the current clinically available therapies work non-specifically to treat hypertrophic scarring, there are few emerging clinical studies being conducted to investigate interventions which target specific pro-inflammatory and pro-fibrotic markers.

## 4 Therapeutic targets for hypertrophic scars

### 4.1 Potential therapeutic targets and strategies

Strategies for reducing aberrant scar formation should center around limiting early inflammatory pro-fibrotic signaling, macrophage-mediated fibroblast-to-myofibroblast differentiation, over-vascularization, and myofibroblast collagen and ECM remodeling.

#### 4.1.1 TGF-β/Smad signaling pathway

**FIGURE 2:**
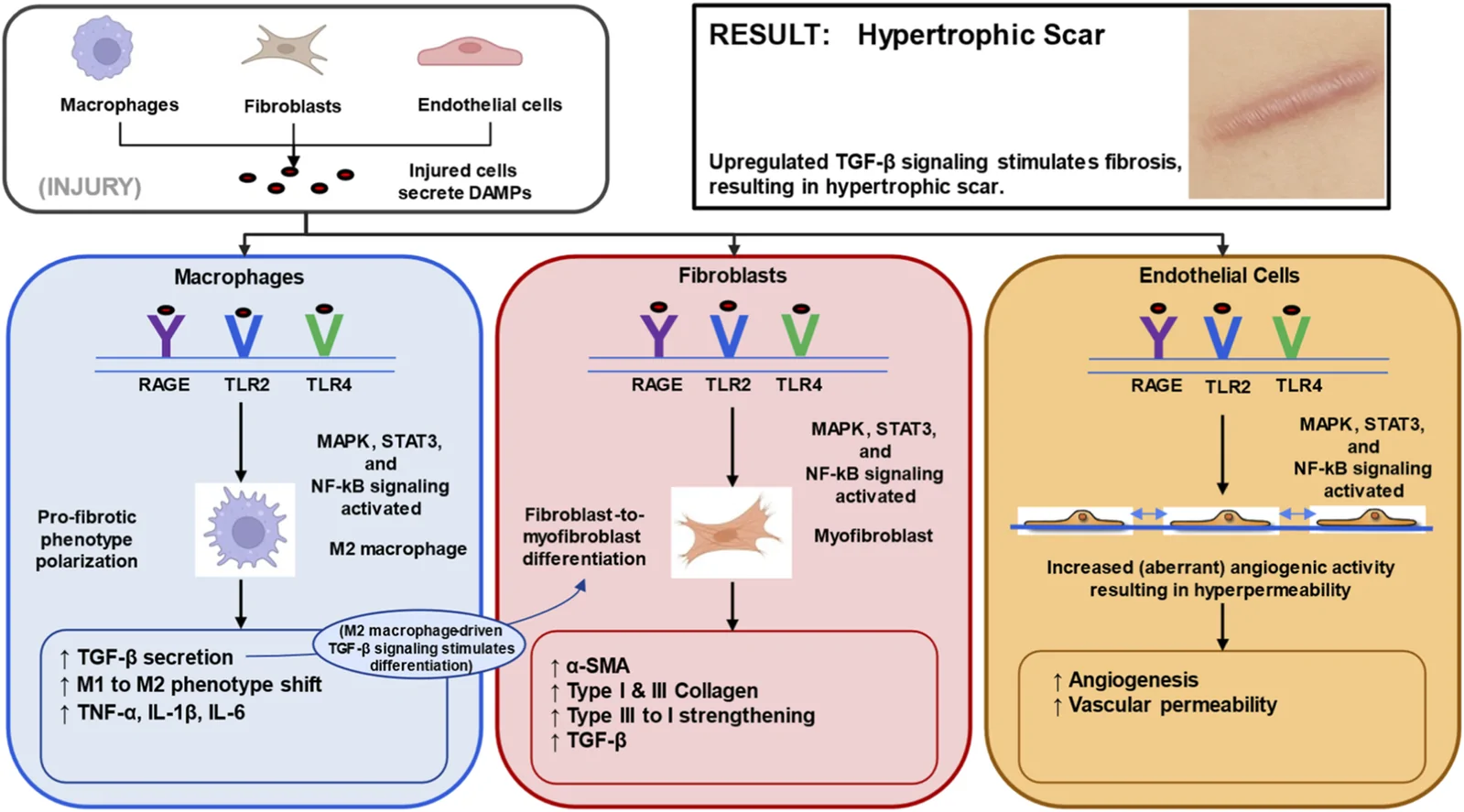
NF-κB, STAT3, and MAPK signaling pathways are stimulated by pro-inflammatory factors and DAMPs leading to over-expression of pro-fibrotic factors, increased vascular permeability and angiogenesis, and macrophage-driven TGF-β signaling leading to fibroblast to myofibroblast differentiation and hypertrophic scar. The release of DAMPs upregulates pro-fibrotic behavior in macrophages, fibroblasts, and endothelial cells as the primary cell types contributing to hypertrophic scar.

TGF-β/Smad signaling regulates scarring and fibrosis, and when disturbed can lead to irregular collagen deposition (higher proportion of collagen I/III, abnormal bundling) and therefore hypertrophic scarring (Lichtman et al., 2016; Tang et al., 2018; Zhang et al., 2020). TGF-β additionally stimulates fibroblast to myofibroblast differentiation during chronic inflammation (Carthy, 2018). Modulating TGF-β/Smad signaling to limit myofibroblast formation and over-deposition of collagen is a frequent target in controlling hypertrophic scar formation (Figure 2).

In human fibrosis explants, it was found that the primary mechanism for fibroblast-to-myofibroblast differentiation in hypertrophic scars was from M2 phenotype macrophages secreting inflammatory factors mediated by the TGF-β/Smad3 and STAT3 pathways. The study found that Smad3 inhibition and macrophage depletion indeed reduced scarring in full thickness skin wounds (Jia et al., 2025). Likewise, another study found that modulating expression of ubiquitin-specific protease 4 (USP4) by transfecting fibroblasts with USP4 siRNA resulted in reduced TGF-β and increased Smad7 expression, and therefore reduced fibroblast proliferation and migration, and increased apoptosis (Huang et al., 2020).

Recent studies therefore suggest that treatments targeting TGF-β/Smad pathways can be an effective method of limiting fibroproliferation and reducing hypertrophic scarring.

#### 4.1.2 NF-κ and STAT3 inflammatory signaling pathways

Given the characteristic prolonged inflammation phase in pathological wounds leading to hypertrophic scars, a common treatment strategy is to target pro-inflammatory cytokines and dampen inflammatory signaling pathways such as nuclear factor kappa B (NF-κB) and STAT3. For example, the NF-κB pathway has been implicated in keloid pathogenesis; it was found that NF-κB signaling was higher in keloid fibroblasts relative to normal fibroblasts following TNF-α treatment, and blocking the signaling with dehydroxymethylepoxyquinomicin (DHMEQ) resulted in decreased fibroblast proliferation and lower type I collagen deposition (Makino et al., 2008; Fujita et al., 2019; Wang Z.-C. et al., 2020). A potential strategy is to target upstream, pro-inflammatory factors such as IL-6, IL-8, or C-X-C motif chemokine ligand 12 (CXCL12) which are known to stimulate NF-κB pathways (Abdou et al., 2014; Zhang et al., 2016; Wang Z.-C. et al., 2020). IL-6 is directly implicated in the activation of STAT3 signaling, as hypertrophic scar fibroblasts have been demonstrated to have enhanced sensitivity to IL-6 trans-signaling and have higher STAT3 expression. The same study found that knockdown of STAT3 attenuated the expression of fibroproliferative c-Myc markers, thereby identifying NF-κB and STAT3 pathways as contributors to hypertrophic scar pathogenesis and targets for scar reduction (Ray et al., 2013).

#### 4.1.3 Anti-angiogenic strategies

Another strategy to reduce hypertrophic scars is to target the vascular component of pathological wound healing. By limiting over-vascularization in the wound space, the release of pro-fibrotic and inflammatory cytokines can be reduced. A recent study found that anti-angiogenic shikonin-based gel treatment significantly decreased the expression of VEGF and α-SMA in L929 fibroblasts (Wang et al., 2025). The same study found that the treatment significantly lowered scar elevation using an *in vivo* rabbit ear scar model relative to untreated scar wounds (Wang et al., 2025). Other studies have used usnic acid in the same rabbit ear model and demonstrated lower scar elevation and significantly inhibited hypertrophic scar angiogenesis by quantifying the density of CD31-positive vasculature in the tissue sections (Song et al., 2018). The study additionally investigated *in vitro* HUVEC migration/tube formation and they found treatment significantly lessened cell migration and tube formation (Song et al., 2018). Another study tested pigment-epithelium-derived factor in the rabbit ear model and saw decreased angiogenesis and decreased collagen deposition at the wound site (Ma et al., 2020). As such, there are various anti-angiogenic therapeutics that limit over-vascularization and reduce pro-fibrotic signaling, making anti-angiogenic factors a valuable target for hypertrophic scarring.

#### 4.1.4 Mechanotransduction

External mechanical forces, particularly stretching at the wound site, are strongly linked to causing hypertrophic scars. It is suggested that hypertrophic and keloid scars are site-specific, developing more often in regions with high tension (Ogawa et al., 2012; Ogawa, 2024). Stretching forces activate mechanoreceptors on immune cells, endothelial cells, and fibroblasts and their ECM components (Ogawa, 2008; 2024). One such mechanotransduction signaling pathway shown to impact stretch-induced hypertrophic scars is integrin-focal adhesion kinase (FAK) signaling, shown to be significantly upregulated in human hypertrophic scar tissue *in vivo* and *in vitro* (Su et al., 2016; Yin et al., 2022). Another example is Wnt/β-catenin signaling pathways, where hypertrophic scars are observed to have elevated β-catenin resulting in fibrosis and cross-signaling between TGF-β pathways (Cheon et al., 2005; Lam and Gottardi, 2011; Bastakoty and Young, 2016; Yin et al., 2022). Other mechanically activated signaling such as Piezo1 are known to promote hypertrophic fibroblast proliferation, motility, and myofibroblast differentiation upon induced mechanical stretching (He et al., 2021). Indeed, mechanical stretching can stimulate fibroblast-to-myofibroblast differentiation through α-SMA, yes-associated protein/transcriptional co-activator with PDZ-binding motif (YAP/TAZ) expression and Hippo/YAP signaling (Aragona et al., 2013; Dupont, 2016; Hinz et al., 2019; D’Urso and Kurniawan, 2020; He et al., 2021).

In clinical contexts, a prevention strategy for hypertrophic scars is wound/scar fixation or using tapes or other stabilizer aids to minimize tension or stretching of the wound (Atkinson et al., 2005). Besides mechanical restriction, various mechanotransducted signaling pathways have been identified as potential therapeutic targets to mitigate stretch-mediated hypertrophic scars.

### 4.2 Limitations of therapeutic approaches

There is limited clinical data available involving these emerging therapeutic targets. Hypertrophic scars are a multifaceted health problem involving numerous dysregulated inflammatory, proliferative, angiogenic, and mechanotransducted signaling pathways. A thematic challenge in hypertrophic scar treatment is the inability to address all the contributing factors in a single therapeutic, thus necessitating a combination of treatments. Regardless, there are some clinically available topical drugs implementing similar therapeutic strategies that show evidence in improving hypertrophic scarring in humans. Namely, retinoids and vitamin A derivative products inhibit TGF-β-induced type I collagen expression in human fibroblasts *in vitro*. Studies have demonstrated treatment with tretinoin significantly decreased the size of human keloid lesions over a 12-week study period and effectively prevented hypertrophic scars at a comparable level to silicone gel treatment (Panabiere-Castaings, 1988; Viera et al., 2010; Kwon et al., 2014). Furthermore, imiquimod has been shown to reduce the recurrence rate of excised human keloids and improve scar outcomes (flattening, pigmentation improvement) (Berman and Kaufman, 2002; Shapiro, 2004; Viera et al., 2010). Imiquimod is a topical immunomodulator that induces the production of anti-fibrotic cytokines such as interferon-alpha (IFN-a), with the potential to prevent or limit fibrotic scar tissue formation (Berman and Kaufman, 2002; Shapiro, 2004). There are limited commercially available drug-based therapeutics for hypertrophic scarring, though what has been seen in clinic prevents or treats hypertrophic scars through the mechanisms summarized (Table 2).

**TABLE 2:** Therapeutic targets and signaling pathways relevant to hypertrophic scarring.

Therapeutic target/strategy	Mechanism of action leading to hypertrophic scar	Summary of strategy	References
TGF-β/Smad	Signaling regulates collagen deposition. TGF-β signaling stimulates fibroblast to myofibroblast differentiation	Inhibit Smad3 and TGF-β expression to limit fibroblast proliferation, migration, and macrophage-mediated myofibroblast differentiation	Huang et al. (2020), Zhang et al. (2020)
NF-κB and STAT3	Signaling regulates inflammatory response in wound	Inhibit pro-inflammatory cytokines such as IL-6, IL-8, CXCL12 known to stimulate NF-κB pathways and reduce fibrotic scar pathogenesis	Zhang et al. (2016), Fujita et al. (2019)
Anti-angiogenic strategies	Overperfusion and overvascularization in wound leads to fibrosis	Anti-angiogenic treatment inhibits overvascularization and delivery of pro-inflammatory cytokines	Song et al. (2018), Ma et al. (2020), Wang et al. (2025)
Mechanotransduction strategies	Stretching and tensile forces stimulate mechanotransducted signaling pathways and pro-fibrotic activity	Stabilize wound space and limit mobility. Target pro-fibrotic cytokines released in mechanotransducted pathways (α-SMA, YAP/TAZ expression and Hippo/YAP signaling)	He et al. (2021), Ogawa (2024)

Several signaling pathways and pro-fibrotic activities have been identified leading to hypertrophic scarring. In continuing the multi-line approach to treating hypertrophic scars, the development of new therapeutics may look at the pro-fibrotic and pro-inflammatory cytokines and signaling activities aforementioned. Development of innovative and novel treatments may focus on targeting the expression of pro-fibrotic and pro-inflammatory cytokines and limiting signaling activity leading to hypertrophic scars.

Clinical data for these therapeutic targets is limited due to challenges in delivery of therapeutic, formulation, and general complexity of hypertrophic scar pathology (see Section 3). As such, further *in vitro, in vivo,* and clinical research is needed to better investigate the effectiveness and translatability of these targets.

## 5 Available models for investigating hypertrophic scars

Research models used to study hypertrophic scars are commonly categorized between 2D and 3D cell culture and select animal models (Mony et al., 2023). Modeling hypertrophic scars is a unique challenge due to the complex pro-inflammatory microenvironment and the “raised” and fibrotic characteristic of the tissue. Thus, *in vitro* models often are limited or require additional design consideration to better represent the hypertrophic scar. Likewise, *in vivo* models require consideration in order to mimic human hypertrophic scar physiology.

### 5.1 2D *in vitro*

2D *in vitro* models of hypertrophic scars typically involve monocultures of representative skin cells (fibroblasts, keratinocytes, and endothelial cells). These cell culture models are most effective for the study of simple cell behaviors that can impact the formation of hypertrophic scars. Common *in vitro* wound assays are scratch assays, which measure cellular migration upon inducing a scratch down the cell monolayer (Grada et al., 2017; Kumar et al., 2024; Chu et al., 2025). Other 2D wound models are stamp, thermal, electrical, and optical injuries which similarly seek to determine the cell ability to migrate across the wound boundary and re-form the monolayer (Urciuolo et al., 2022). The rate of reepithelialization and density of cells during the assay could be used to make conclusions on hypertrophic scar development, e.g., rapid reepithelialization, overly dense monolayer, and polarity of fibroblasts could suggest pro-fibrotic stimulation (A. DiPietro L. and Schrementi, 2018; Urciuolo et al., 2022). Other experimental options are cell proliferation assays, for which overproliferative activity is implicated in hypertrophic scarring (Nabai et al., 2020). Another suggested assay is tube formation assay, given that hypertrophic scarring is associated with overvascularization in the wound (Molina et al., 2022).

2D cell culture models are limited in complexity and are generally only able to investigate few cellular responses (migration, proliferation, angiogenesis, etc.) pertaining to hypertrophic scars. As a result, these models do not capture the multicellular response and the various cross-signaling activity that in total contribute to the formation of hypertrophic scars, and it is recommended to investigate additional 3D *in vitro* or *in vivo* models.

### 5.2 3D *in vitro*

3D *in vitro* models can better represent ECM components and can support cocultures of cells to create a more robust representation of hypertrophic scars. 3D exogeneous scaffolds can be seeded with fibroblasts or a co-culture with keratinocytes or endothelial cells (Urciuolo et al., 2022). Such scaffolds are most commonly constructed using rat tail collagen type I containing human fibroblasts (Urciuolo et al., 2022). A study tested the contraction of a 3D rat tail collagen type I construct seeded with fibroblasts post-treatment with pro-fibrotic factors (TGF-β, IL-6, IL-8) to represent the hypertrophic scar environment and found significantly greater contraction relative to untreated scaffolds (Chawla and Ghosh, 2018). This is a similar refinement on the original fibroblast-populated collagen lattice (FPCL) model which extracted rat tail collagen type I to form 3D constructs to be seeded with fibroblasts and underwent contraction measurement (Bell et al., 1979). These collagen gel constructs have the potential to support multiple cell types. A study sought to observe the dynamics of endothelial and fibroblast cells in a 3D wound environment by a co-culture of HUVECs and human lung fibroblasts (HLFs) seeded in a similar collagen gel. The study quantified the 3D cell migration post full-thickness injury, proposing a more representative model of the ECM and the dynamic wound environment (Tefft et al., 2021). Likewise, similar methods have been used to create 3D constructs of keratinocytes and fibroblasts co-cultures (Carlson et al., 2008).

While collagen-based matrices and scaffolds are the most common method of constructing a 3D cell model for hypertrophic scars, there have been recent studies developing alternative models. These newer 3D models seek to improve the original FPCL model by using patient-derived fibroblasts, incorporating full-thickness models with immune cells, and more accessible microfluidic device models. One study developed a 3D function hypertrophic scar model by bioprinting preformed cellular aggregates made of decellularized scar ECM and pre-cultured patient-derived fibroblasts (Bin et al., 2022). The study found that the aggregates displayed functional scar tissue self-organization and expressed pro-fibrotic markers (α-SMA, TGF-β), suggesting the model could simulate tissue formation and alignment as well as express representative hypertrophic scar markers (Bin et al., 2022). Other innovations include using 3D-organotypic skin cultures embedded with normal or hypertrophic scar-derived fibroblasts, incorporating adipose tissue to represent deep wounds, burn-induced hypertrophic scarring (Raktoe et al., 2023). The full-thickness model was developed using layers of collagen gel containing adipose tissue, a cell-free layer, and a normal or hypertrophic scar-derived fibroblast-embedded layer. The model underwent thermal wounding and re-epithelialization of the full-thickness model was measured. Similar full-thickness models incorporated macrophages, T cells, keratinocytes, and fibroblasts to better represent the immune component of hypertrophic scars (macrophage-to-myofibroblast signaling) (Pupovac et al., 2018). There have also been device-based approaches to modeling hypertrophic scarring, such as a skin-on-chip microfluidic device consisting of multiple sheets of polydimethylsiloxane cultured with keratinocytes, fibroblasts, and endothelial cells on each layer, or other microfluidics cultured additionally with macrophages (Wufuer et al., 2016; Biglari et al., 2019; Urciuolo et al., 2022).

The full-thickness models aforementioned have the ability to simulate the interplay between various skin cell and immune cell types. Limitations such as low nutrient transport due to lack of flow are addressed in microfluidic models. The greatest potential limitation of collagen-based constructs is likely the lack of external mechanical forces. Mechanical stretching and tension on the wound are significant contributors of hypertrophic scar development, and the external forces are responsible for stimulating several mechanotransducted signaling pathways. This could be partially simulated through inducing a similar microenvironment by treating with relevant cytokines such as α-SMA or β-catenin such that integrins/FAK, Wnt/β-catenin, YAP/TAZ, and Hippo/YAP signaling pathways are activated. Additionally, there are strain systems used to apply mechanical tension *in vitro* such as the *FX-4000™ Flexcell® Tension System* which was used in studies to apply uniaxial and cyclic strain in fibroblast wound healing assays (Rolin et al., 2014). However, the complexity of these systems is generally limited to uniaxial stretching and may not capture all the local forces (shear, multiaxial tension, compression, etc.) that typical wounds experience. To best represent the combined dynamic wound microenvironment and variable mechanical stimuli observed in human scarring, hypertrophic scarring may best be modeled *in vivo*.

### 5.3 Animal models

*In vivo* models of hypertrophic scars are limited in their ability to fully reproduce the pathology and structure of human hypertrophic scars. The rabbit ear model is most commonly used as it produces raised scars resembling human hypertrophic scars (Xiang et al., 2004; Song et al., 2023). Wounding is performed by inducing surgical full skin thickness defects on the ventral side of the ear (Xiang et al., 2004; Diao et al., 2013). This method of wounding is most common, though other thermal burn and cryosurgery methods are novelly used to mimic hypertrophic scarring in the rabbit ear (Friedrich et al., 2017; Tunca et al., 2019; Mony et al., 2023).

Mimicking hypertrophic scars in mice or other rodent models is uniquely challenging as wound healing and scar development differ significantly between mice and humans due to rapid contraction of rodent panniculus carnosus muscles (Naldaiz-Gastesi et al., 2018; Maden and Brant, 2019). To better simulate human hypertrophic scar development, various mechanical strategies such as wound splinting or applying pressure and tensile forces throughout the study duration are used and have resulted in more hypertrophic scar features (Jimi et al., 2021; Son and Hinz, 2021). Applying mechanical force to rodent wounds results in production of scar tissue and the splint can be used as a reservoir for treatment to investigate scar development (Son and Hinz, 2021).

Lastly, porcine models may be most representative of human scarring due to anatomical similarities and full-thickness wounds. The scar is formed through either full-thickness excisional wounds or deep-burn wounds, creating a full-thickness scar resembling human hypertrophic scars in contractility and wound healing processes (Cuttle et al., 2006; Singer and McClain, 2006; Seaton et al., 2015; Deng et al., 2018). Porcine scar models are typically considered to be the gold standard but are generally limited due to complexity of handling, sample size, and study duration (Seaton et al., 2015).

Animal models for hypertrophic scars are limited, and no single model fully captures the complexity of human hypertrophic scars. While rodent models are favored for their low cost and accessibility, rodent wound healing mechanics occur through contraction of the panniculus carnosus muscle as opposed to human granulation tissue generation and re-epithelialization (Chen et al., 2013; Zhang et al., 2026). Subsequently, rodent scars lack the highly raised and fibrotic characteristics that are of interest in hypertrophic scars (Rössler et al., 2022). The rabbit ear model produces raised scars, though the organization of collagen fibers does not match the nature of human hypertrophic or keloid scars (Marttala et al., 2016). Furthermore, the model notably lacks mechanical tension and stretching forces that are characteristic in the formation of hypertrophic scars (Cao et al., 2022). Though porcine models are most representative physiologically of human skin and can produce fibrotic scars, porcine studies are limited by high cost and accessibility (Seaton et al., 2015). Human hypertrophic scars are influenced by anatomical location and mechanical tension, and factors such as age, disease, and genetics cannot be fully captured by animal models.

Reproducing hypertrophic scars in small animal models is a unique challenge due to differences in wound healing physiology, often requiring niche anatomy (rabbit ear) or additional equipment (rodent splints) at the cost of translatability. While porcine models are better representative of human hypertrophic scars, large animal models present additional challenges. As such, additional research is needed in developing adequate *in vitro* and *in vivo* models of hypertrophic scars.

## 6 Translational applications for other scar types

While hypertrophic and keloid scars are a concern for deep wounds, there are other scar outcomes that can develop such as atrophic scars from acne, scars formed post-spinal cord injury (SCI), and diabetic and pressure ulcers. The presentation of other scarring can vary from mechanically weak scars with poor ECM organization to more fibrotic and variably dense tissue. These other scar types originate from pathological wound healing, dysfunctional inflammatory signaling, and excessive fibroblast activation.

### 6.1 SCI-induced pressure wounds and diabetic foot ulcers

One of the most frequent complications of SCI is pressure wounds, which form due to immobility and prolonged pressure on bony prominences. Sustained pressure over particularly bony prominences such as the spinal cord result in tissue hypoxia, leading to ischemia and necrosis (Vecin and Gater, 2022; Zaidi and Sharma, 2026). These wounds have a high recurrence rate as the integrity of the tissue and scar are mechanically weak and easily compromised (Zaidi and Sharma, 2026).

While hypertrophic scars are characterized by highly dense ECM networks and fibrotic tissue, pressure ulcer scars conversely express decreased fibronectin, decreased blood flow and vascularization, and a lower proportion of type I collagen to type III collagen (Rappl, 2008). Additionally, conditions such as diabetes mellitus resulting in decreased neutrophil and macrophage activation, decreased VEGF and TNF-α expression, and lower fibroblast proliferation greatly increased risk of pressure wound formation (Kruger et al., 2013; Avishai et al., 2017; Semadi, 2019). The lack of inflammatory signaling leads to a decreased fibroblast population and under-expression of contractile markers, resulting in a weaker scar.

The investigation of SCI-induced pressure wounds is complex, with limited *in vitro* and *in vivo* models. Notably, a mouse model has been developed for SCI pressure wounds that shows delayed wound healing, larger scar area, and lower distributions of key scar contraction markers (Kumar et al., 2018; 2019; 2026). In this model, SCI was induced transection between the T9 and T10 vertebra. Pressure wounds were induced below the site of SCI by sandwiching a skin fold on the back of the mouse between two magnetic discs (Kumar et al., 2019). Following this model, it was demonstrated that the epidermis in SCI-induced pressure wound mice was significantly reduced and wound closure was significantly slower. Further, the scar area was significantly larger than non-SCI mice. The distribution of α-SMA, a key marker of myofibroblast formation and tissue contraction in the TGF-β pathway, was observed to be significantly less present in pressure wound SCI groups (Kumar et al., 2019). This suggests that the final SCI-induced pressure wound scar is larger in area but may have less structural integrity. Using this model, it has also been demonstrated that treatment can improve scar outcomes, as treatment with coacervates made of elastin-like polypeptides fused with melanocyte-stimulating hormone and monocyte chemoattractant protein increased epidermal and dermal thickness, reduced scar area, and increased α-SMA expression and macrophage migration (Kumar et al., 2026).

Diabetic ulcers similarly form on bony prominences, particularly the foot due to peripheral neuropathy, peripheral arterial disease, irregular vascularization, and dysregulated wound healing processes (Srinivas-Shankar et al., 2026). Diabetic wounds are characterized by an extended inflammatory phase of healing with little angiogenic activity, resulting in low wound contraction and mechanically weak scar tissue (Dasari et al., 2021). Pressure wounds and diabetic foot ulcer scars both suffer high recurrence risk due to the insufficient collagen and ECM produced by low fibrotic activity.

### 6.2 Atrophic scars

Atrophic scars are characteristically depressed with decreased epidermal thickness due to less fibrotic activity and poor collagen fiber density (Kohlhauser et al., 2024). The primary mechanism resulting in atrophic scars is degradation of collagen due to elevated levels of MMP-1, MMP-2, MMP-3, MMP-9, and MMP-12 (Kang et al., 2005; Choi et al., 2008; Ray Jalian et al., 2008; Kohlhauser et al., 2024). The overexpression of MMPs degrades collagen fibers, resulting in a loose ECM and mechanically weak scar. It is suggested that aberrant TGF-β signaling is, like hypertrophic scarring, the modulator for pathogenesis of atrophic scars (Moon et al., 2019).

The formation of pathological scars has been observed to be caused by a combination of dysregulated inflammatory signaling, fibroblast activity, contractile and collagen-production marker expression. Hypertrophic scars are an example of extreme fibrosis, whereas scars from chronic wounds (pressure ulcers due to SCI, diabetic foot ulcers) have less mechanical integrity due to poor ECM deposition. While the clinical presentation may differ, pathological scars stem from similar mechanisms of inflammation and fibroblast activation. The investigation of relevant inflammatory and mechanotransducted signaling pathways can be applied to the study of both over-fibrotic scars as well as mechanically weak scars, such as those formed from pressure wounds post-SCI.

### 6.3 Modulating scar-signaling to improve pathologic scar outcomes

Pathologic scars range from mechanically weak and under-fibrotic scars, such as SCI-induced pressure wound scars, to overly dense and fibrotic hypertrophic scars. These scar types share similar inflammatory signaling dysfunction, and the mechanistic findings from hypertrophic scar research can be used to modulate scar biology and alter the mechanical strength of the tissue. In the case of hypertrophic scars, excessive TGF-β, Smad-3, NF-κB, Wnt/β-catenin, FAK, YAP/TAZ, and integrin-mediated mechanotransduction signaling result in collagen over-deposition. Conversely, modulating the same pathways is a therapeutic strategy for restoring tissue integrity in mechanically weak scars. Stimulating TGF-β, Smad-3, and NF-κB pathways may improve collagen deposition in the case of SCI pressure wound scars and atrophic scars, thereby having a regenerative or therapeutic effect. Likewise, suppressing these signaling pathways can reduce pro-fibrotic activity to treat hypertrophic scars. Modulating the identified signaling pathways can be used to optimize the fibrotic response and improve pathologic scar outcomes at both under and over-fibrotic scar extremes.

## 7 Conclusion and future prospectives

Hypertrophic scar development is the result of a compounding of pro-fibrotic, inflammatory, and angiogenic signaling exacerbated by mechanical forces throughout the phases of pathological wound healing. Excessive inflammatory cytokines and over-vascularization of the wound stimulate myofibroblast differentiation, primarily through fibroblast-to-myofibroblast pathways, resulting in overproduction of collagen type I and type III. Hypertrophic scars tend to be site-specific, occurring in regions of high movement and stretching, implicating the important role of mechanotransduction signaling in fibrosis. Focusing on these primary pathways are potential targets for therapeutic development. Methods of investigating these mechanisms of hypertrophic scar include 2D and 3D *in vitro* techniques and limited animal models. 2D *in vitro* models consist of a monoculture to be assessed in simple wound healing assays, whereas 3D models can incorporate multiple cell types and better replicate the ECM. Lastly, rabbit ear models are the most frequent *in vivo* model of hypertrophic scarring, and efforts to make rodent models more representative through mechanical manipulation and tension can partially replicate human hypertrophic scarring.

Several recent clinical trial studies assessed lasers, compression, and negative pressure interventions, consistent with the clinically available therapies for hypertrophic scars. Few studies involved drug interventions that targeted the inflammatory signaling mechanisms leading to hypertrophic scars. The mechanisms behind fibrotic scar tissue generation are excessive inflammatory and TGF-β signaling and mechanotransduction, as these factors stimulates fibroblast-to-myofibroblast differentiation and collagen production. Future prospectives for hypertrophic scar research should seek to investigate interventions which disrupt the early inflammatory signaling responsible for hypertrophic scarring. This research can be translated to other pathological scar types, such as SCI-induced pressure wounds which inversely lack TGF-β and pro-fibrotic signaling activity.
