# Supplementary material for: Current therapeutic strategies and available research models for investigating hypertrophic scars
Source: Front Pharmacol. 2026 Sep 9;17:1887685. doi: 10.3389/fphar.2026.1887685 (PMC13598706; doi:10.3389/fphar.2026.1887685)
Supplement: Supplementary file 1 [file Table1.docx]

Supplementary Material

**Supplementary Table 1.** Recently completed hypertrophic scar clinical trial studies

| **NCT Number** | **Study Title** | **Phase (#)** | **Enrolled (#)** | **Initial Dose / Repeated Dose** | **Frequency of Repetition** | **Remarks** | **Sponsor** | **References** |
| --- | --- | --- | --- | --- | --- | --- | --- | --- |
| NCT04877756 | Study to Evaluate Efficacy of OLX10010 in Reducing Recurrence of Hypertrophic Scarring After Scar Revision Surgery | Phase 2a | 22 | Arm A: 2.0 mg/cm OLX10010  Arm B: 5.0 mg/cm OLX10010 | Biweekly (every two weeks), for a total of six doses | No results | Olix Pharmaceuticals, Inc. |  |
| NCT06347081 | Effect of Nd-YAG Laser on Hypertrophic Scar | N/A | 40 | Nd-YAG laser at 532nm | Biweekly, for a total of 14 sessions | Significant differences observed in scar volume and  Vancouver Scar Scale scores after treatment in study group | Cairo University | (Attalla et al., 2025) |
| NCT07360483 | Treatment of Hypertrophic Scars Using Fractional CO2 Laser Alone in Comparison With Adding Botulinum Toxin Either Through Intralesional Injection or Assisted Drug Delivery | N/A | 48 | DEKA SmartXide DOT laser: power (18-20W), dwell time (800-1000µs), spacing (500-600µm, 13% density), micro spot size (120µm), and 2-3 stacks  2.5 U/cm³ intralesional Botulism Toxin | Once a month for two months | The current study aimed to evaluate the effectiveness of fractional laser treatment alone and fractional laser combined with botulinum toxin A, delivered either through injections or topical application fractional laser-assisted drug delivery (FLADD) in hypertrophic burn scar | Cairo University |  |
| NCT04769089 | Effects of Pulsed Dye and CO2 Laser in Treatment of Hypertrophic Burn Scars. | N/A | 25 | Pulse Dye Laser: Duration of 0.45ms, and fluence of 5.25J/s.  CO2 Laser:  300Hz, 15mJ, 15% density. | Three treatments, 4-6 weeks apart | Hypertrophic burn scars treated with CO2 or PDL showed a significant decrease in Patient and Observer Scar Assessment Scale score | The Metis Foundation | (Cooper et al., 2022) |
| NCT06690749 | Using Skin Blood Flow to Measure the Effect of Different Doses of Negative Pressure Therapy | N/A | 36 | -105 mmHg, -125 mmHg, and -145 mmHg | Single treatment | -105 mmHg dosage can effectively increase skin blood flow. | Asia University | (Shen et al., 2025) |
| NCT04702529 | Treatment of Fibrotic Scars With Rapid Acoustic Pulse (RAP) Device | N/A | 20 | Single RAP treatment | Biweekly for a total of 3 treatments | No results | Soliton |  |
| NCT06909812 | Silicone vs Pirfenidone in the Treatment of Hypertrophic Scars and Keloids | Phase 3 | 40 | Topical 8% pirfenidone gel  Topical silicone gel | Daily with follow-up at 2, 4, 8, 12, and 16 weeks | No results | Fundacion Nacional para la Ensenanza y la Investigacion de la Dermatologi­a A.C. |  |
| NCT07263100 | Comparative Study Between Intralesional Corticosteroid Injection Alone and Combined Intralesional Corticosteroid Injection With Either Long-pluse Nd YAG or Erbium Yag for Treating Hypertrophic or Keloid Scars | N/A | 20 | TAC injection:  0.1 ml per cm^2^  Nd:YAG laser:  6 passes at a fluence of 11 J/cm2, spot size 3 mm, pulse duration of 10ns, and frequency of 3 Hz. | 5 sessions scheduled 4 weeks apart | No results | Somdech Phra Nangchao Sirikit Hospital |  |
| NCT07209891 | COMPARISON STUDY OF KINESIOTAPE AND LOW-LEVEL LASER THERAPY ON HYPERTROPHIC SCAR | N/A | 60 | LLLT:  905-nm pulsed device at ~16.2 J/cm²  Kinesio tape | 3x/week for 8 weeks | No results | Cairo University |  |
| NCT05429411 | Nonsilicone Gel Sheet for Burn Hypertrophic Scars | N/A | 49 | Silicone gel | 21+ hours as possible | No results | Centre hospitalier de l'UniversitÃ© de MontrÃ©al (CHUM) |  |
| NCT07196358 | Topical Heparin for Prevention of Hypertrophic Scarring in Split-Thickness Skin Graft Donor Sites | N/A | 400 | Gauze soaked in 500 IU/ml heparin | 10 days | Throughout the study period pain was significantly lower in heparin group than control group. At second month and third month, occurrence of hypertrophic scarring frequency was significantly higher in control group than heparin group. | Dr. Ruth K.M. Pfau Civil Hospital, Karachi | (Department of Plastic & Reconstructive Surgery, Dr. Ruth K.M. Pfau Civil Hospital, Karachi, Pakistan. et al., 2025) |
| NCT04812626 | Intralesional Triamcinolone Acetonide and Its Combination With 5-fluorouracil in Keloids and Hypertrophic Scars | N/A | 66 | Triamcinolone acetonide:  0.125ml intralesional  5-fluorouracil:  1ml of 50 mg/ml | Biweekly (every 2 weeks) for total of 6 sessions | The ≥50% reduction in height, reduction in the VSS and POSAS scores, and good to excellent subjective improvement reported by both the patients and the observer were significantly greater in the combination group compared to TAC alone group. The response rate was faster and complications were lesser in the combination group as compared to TAC alone group. | B.P. Koirala Institute of Health Sciences | (Acharya et al., 2024) |
| NCT04951869 | : Protescal: Prevention of Hypertrophic Scar and Keloid Formation Post Caesarean Section | Phase 2  Phase 3 | 90 | Protescal gel (0.5 mL) | Single treatment | No results | Universiti Kebangsaan Malaysia Medical Centre |  |
| NCT05412745 | Class I Medical Device on Post-surgical Scars | N/A | 64 | Kaloidon Plus topical application | Twice daily for 12 weeks | The topical application of a pullulan-based medical device containing *Allium cepa* and HA showed a clear reduction in the local inflammation, which might lead to a reduced probability of developing hypertrophic scars or keloids. | University of Rome Tor Vergata | (Cosio et al., 2023) |
| NCT06198283 | Effects of Pressure Garments on Hypertrophic Hand Scar in Burn Children | N/A | 26 | Pressure garment 8-10 hours/day  LLLT (422-800nm) 3 days/week | 6 weeks | No results | Riphah International University |  |
| NCT06301178 | Effect of Vitamin D Injection on Hypertrophic Scars and Keloids | Phase 1  Phase 2 | 35 | Cholecalciferol:  200,000 IU | Once monthly for 3 months | Injection of vitamin on hypertrophic scars and keloids enhances vascularity and pliability in patients with sufficient serum vitamin D levels and those with deficient or insufficient serum vitamin D levels after improving them by systemic injection of vitamin D without any effect on height and pigmentation of scars. | Mostafa Bahaa | (Hassan et al., 2025) |
| NCT06174155 | Comparative Study Between Botulinum Toxin-A Injection and Shock Waves on Hypertrophic Scars in Hand-burned Children | N/A | 60 | Zimmer enPuls, EMS physio Ltd, Germany | 3 months | No results | Cairo University |  |
| NCT07034482 | Efficacy and Safety of Intralesional 5-Fluorouracil and Triamcinolone Acetonide in Keloids and Hypertrophic Scars | N/A | 41 | 5 mg triamcinolone acetonide injected intralesionally  45 mg 5-FU injected intralesionally | Every week for 6 weeks or until the scar flattened | No results | Maharajgunj Medical Campus |  |
| NCT05128383 | Study to Assess Efficacy and Safety of Dupilumab in the Treatment of Keloids | Phase 2 | 20 | Dupilumab subcutaneous injection  600 mg at initial visit | 600 mg at initial visit and 300mg every 2 weeks until week 22 | No results | Beth Israel Deaconess Medical Center |  |

References

Acharya, R., Agrawal, S., Khadka, D. K., and Pant, A. R. (2024). Efficacy and Safety of Intralesional Triamcinolone Acetonide Alone and its Combination with 5- Fluorouracil in Keloids and Hypertrophic Scars: Randomized, Parallel Group, and Double Blinded Trial. Skin Health and Disease 4, ski2.450. doi: 10.1002/ski2.450

Attalla, A. F., Elsayed, S. E. B., Taha, T. S. E., and Abbas, N. A. (2025). Efficacy of Long-Pulsed 1064 nm Nd:YAG Laser for Hypertrophic Scars: A randomized controlled trial. Egyptian Journal of Physical Therapy and Rehabilitation 0, 0 -0. doi: 10.21608/ejptr.2025.351342.1014

Cooper, J. G., Sicard, D., Sharma, S., Van Gulden, S., McGuire, T. L., Cajiao, M. P., et al. (2020). Spinal Cord Injury Results in Chronic Mechanical Stiffening. J Neurotrauma 37, 494 -506. doi: 10.1089/neu.2019.6540

Cosio, T., Costanza, G., Coniglione, F., Romeo, A., Iacovelli, F., Diluvio, L., et al. (2023). From In Silico Simulation between TGF-β Receptors and Quercetin to Clinical Insight of a Medical Device Containing Allium cepa: Its Efficacy and Tolerability on Post-Surgical Scars. Life 13, 1781. doi: 10.3390/life13081781

Hassan, A. R., Binsaleh, A. Y., El‐Tahlawi, S. M., El‐Amir, A. M., Ishak, M. M., Alsubaie, N., et al. (2025). Impact of Vitamin D Injection on Keloids and Hypertrophic Scars. J of Cosmetic Dermatology 24, e70118. doi: 10.1111/jocd.70118

Lin, X., and Lai, Y. (2024). Scarring Skin: Mechanisms and Therapies. Int J Mol Sci 25, 1458. doi: 10.3390/ijms25031458

Shen, W.-C., Cheng, H.-T., Jan, Y.-K., Liau, B.-Y., Lien, C.-M., Bau, J.-G., et al. (2025). Effect of Negative Pressure Therapy on Skin Blood Flow Responses in Scar Tissue. Cureus 17, e90436. doi: 10.7759/cureus.90436
